# Chronic Exposure to Deoxynivalenol Has No Influence on the Oral Bioavailability of Fumonisin B_1_ in Broiler Chickens

**DOI:** 10.3390/toxins7020560

**Published:** 2015-02-16

**Authors:** Gunther Antonissen, Mathias Devreese, Filip Van Immerseel, Siegrid De Baere, Sabine Hessenberger, An Martel, Siska Croubels

**Affiliations:** 1 Department of Pharmacology, Toxicology and Biochemistry, Faculty of Veterinary Medicine, Ghent University, Salisburylaan 133, Merelbeke 9820, Belgium; E-Mails: Mathias.Devreese@ugent.be (M.D.); Siegrid.DeBaere@ugent.be (S.D.B.); Siska.Croubels@ugent.be (S.C.); 2 Department of Pathology, Bacteriology and Avian Diseases, Faculty of Veterinary Medicine, Ghent University, Salisburylaan 133, Merelbeke 9820, Belgium; E-Mails: Filip.VanImmerseel@ugent.be (F.V.I.); An.Martel@ugent.be (A.M.); 3 Biomin Research Center, Technopark 1, Tulln 3430, Austria; E-Mail: Sabine.Hessenberger@biomin.net

**Keywords:** *Fusarium* toxins, deoxynivalenol, fumonisin B_1_, broiler chickens, toxicokinetics, oral bioavailability

## Abstract

Both deoxynivalenol (DON) and fumonisin B_1_ (FB_1_) are common contaminants of feed. Fumonisins (FBs) in general have a very limited oral bioavailability in healthy animals. Previous studies have demonstrated that chronic exposure to DON impairs the intestinal barrier function and integrity, by affecting the intestinal surface area and function of the tight junctions. This might influence the oral bioavailability of FB_1_, and possibly lead to altered toxicity of this mycotoxin. A toxicokinetic study was performed with two groups of 6 broiler chickens, which were all administered an oral bolus of 2.5 mg FBs/kg BW after three-week exposure to either uncontaminated feed (group 1) or feed contaminated with 3.12 mg DON/kg feed (group 2). No significant differences in toxicokinetic parameters of FB_1_ could be demonstrated between the groups. Also, no increased or decreased body exposure to FB_1_ was observed, since the relative oral bioavailability of FB_1_ after chronic DON exposure was 92.2%.

## 1. Introduction

Mycotoxins are a structurally diverse group of secondary metabolites produced by several fungal genera [1]. Molds belonging to the *Fusarium* genus are commonly affecting feed and food in climatological moderate regions [2]. A worldwide survey on the occurrence and contamination levels of mycotoxins in finished feed for poultry, swine and dairy cows, and feed raw materials indicate that the fusariotoxins deoxynivalenol (DON) and fumonisins (FBs) are the most frequently detected mycotoxins, respectively contaminating 55% and 54% of the 17,316 investigated samples [3]. However, taking into consideration that mycotoxigenic fungi are usually capable of producing more than one mycotoxin, and that feed raw materials are commonly infected with various fungal species at a time, it is very common for feed commodities to be contaminated with different mycotoxins. A study of Streit *et al.* [3], reported that in 53% of the contaminated samples more than one mycotoxin was detected. The final mycotoxin profile of compound feed is also influenced by the levels of the different feed raw materials [4].

The intestinal tract acts as a dynamic barrier, which regulates the entry of foreign antigens into the underlying tissues including food proteins, xenobiotics (such as drugs and mycotoxins), commensal microbiota and pathogens [5]. Following the oral intake of mycotoxin-contaminated feed, the intestinal epithelium will be exposed to high concentrations of mycotoxins [5,6]. Since the main toxic effect of DON at the cellular level is the inhibition of protein synthesis, rapidly proliferating cells in tissues with a high protein turnover, such as the small intestine, are most affected [7]. Several studies demonstrated a negative effect of DON on the intestinal morphology. DON decreases the total intestinal absorption surface area for nutrients by reducing the villus height and crypt depth [8,9,10,11]. Furthermore, several *in vitro* and *in vivo* studies reported that DON alters the intestinal epithelial integrity and permeability, by affecting the function of the tight junctions [8,9,12]. As a result of the negative impact of DON on the intestinal integrity, DON is able to increase the translocation of septicemic *E*. *coli* and increase the permeability to doxycycline and paromomycin over porcine intestinal epithelial cell monolayers [12,13].

As stated above, in addition to DON, FBs are ubiquitous contaminants of corn and other grain products. FBs are produced by *Fusarium verticillioides*, *F. proliferatum*, and other *Fusarium* species [14]. More than 28 fumonisin homologues have been described, with fumonisin B_1_ (FB_1_) as the most thoroughly investigated because of its frequent occurrence and toxicological importance. Fumonisin B_2_ (FB_2_), FB_3_ and FB_4_ are less prevalent, and are structurally different from FB_1_ in the number and position of hydroxyl groups [14,15]. FBs mainly act by inhibiting sphinganine *N*-acyl transferase and consequently disrupt the ceramide and sphingolipid metabolism [16]. Liver, kidneys and the intestinal tract are target organs of FBs toxicity in most animal species [14,17,18]. However, species-specific differences exist in the main affected organs. In horses, FB_1_ mainly affects the brain inducing leukoencephalomalacia, while in pigs the heart and lungs are the most important target organs of FB_1_, causing pulmonary edema [14]. Poultry are often considered to be quite resistant toward the deleterious effects of FBs, although important differences are observed depending on the age [19] and species [20,21,22,23,24]. Increased mortality due to FB_1_ has only been demonstrated in broiler chicks during the first three days of life (≥125 mg/kg feed) [19] and in growing ducks of 12–14 weeks old (20 mg/kg feed) [22]. No mortality has been recorded in laying hens, turkeys or older broiler chickens fed high doses of FB_1_ (≥200 mg/kg feed) for several weeks [20,21,23,25]. Moreover, it has been shown that FBs can reduce growth performance, and induce alterations in serum constituents and enzyme activities demonstrating hepatic toxicity in broilers, turkeys and ducks [20,21,22,24,25,26,27,28].

In different animal species it is shown that FBs are absorbed very poorly after oral administration. Vudathula *et al.* [29] showed an oral bioavailability (F) of 0.71% in laying hens administered 2 mg [14C]FB_1_/kg bodyweight (BW). In turkeys and ducks, a similar F was demonstrated after administering 100 mg FB_1_/kg BW, namely 2.0%–2.3% and 3.2%, respectively [27,28]. Benlashehr *et al.* [30] demonstrated that the toxicokinetics parameters of FB_2_ are not strongly different from these of FB_1_ in ducks and turkeys. Furthermore, the intestinal absorption of FBs in avian species is comparable with mammalian species [31,32,33]. This poor intestinal absorption of FBs has been appointed as the “fumonisin paradox” by Shier [34], or how a toxin can induce liver failure in poultry although it is not effectively absorbed after oral intake. Because the mycotoxins DON and FBs frequently co-occur, and taken into account that FBs have a low oral bioavailability in healthy animals and DON impairs the intestinal barrier and/or decreases the total intestinal absorption surface area, the aim of this study was to investigate whether chronic exposure to DON could influence the intestinal absorption of FBs leading to an altered exposure and increased toxic effects of this mycotoxin in broiler chickens. Because FB_1_ is the most abundant of the FBs in feed, and toxicokinetics parameters of FB_1_ en FB_2_ are strongly similar [30], the impact of DON on the toxicokinetics parameters of FB_1_ was investigated. 

## 2. Results and Discussion

No significant effects on BW or feed intake were seen after chronic exposure to DON (data not shown). For each diet, control and DON contaminated, no macroscopic lesions were found during gross postmortem examination. 

**Figure 1:**
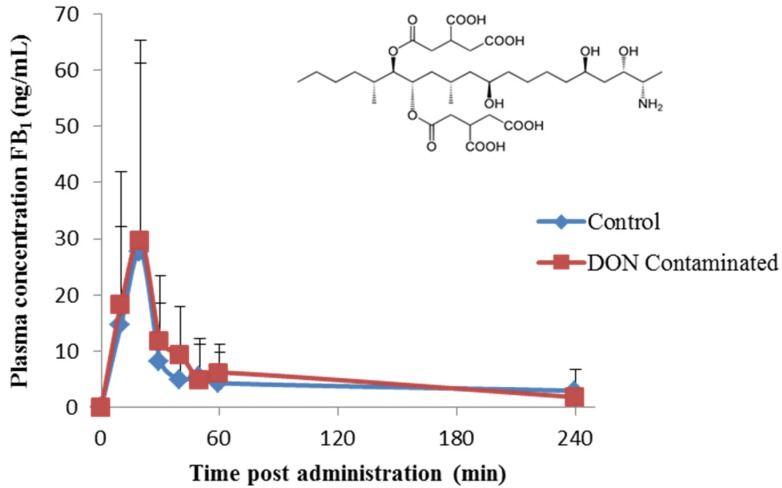
Chemical structure (insert) and plasma concentration-time profile of fumonisin B_1_ (FB_1_) administered as a single oral bolus of fumonisins to broiler chickens (2.5 mg FBs/kg BW, *n* = 6), after 3 weeks exposure to either a diet contaminated with deoxynivalenol (DON contaminated, contamination level: 3.12 mg DON/kg feed) or uncontaminated (control) feed. Values are presented as mean + SD.

After a single oral bolus administration of 2.5 mg FBs/kg BW (1.91 mg FB_1_ and 0.59 mg FB_2_), quantifiable plasma concentrations of FB_1_ were detected (Figure 1). The dose was calculated based on the European maximum guidance level of 20 mg FB_1_ + FB_2_/kg feed [35] and the daily feed intake of the birds (125 g/kg BW). As shown in Figure 1, the plasma concentration-time profile revealed that FB_1_ reached the maximum plasma concentration (T_max_) at 20 min after oral dosing in both control and DON contaminated group. This rapid appearance of FB_1_ in the systemic circulation indicates that the ingested toxin is absorbed mainly in the proximal part of the intestinal tract. The T_max_ was reached more rapidly compared to studies in layers, turkeys and ducks, where a T_max_ of 60 min, 180 min and 60–120 min has been described, respectively [27,28,29]. This difference might be induced by feed deprivation prior to oral FB_1_ administration in the present study, whereas in the other studies feed was not deprived. The delaying effect of feed on absorption of mycotoxins has previously been described for DON in pigs with a T_max_ of 1.3 h and 4.1 h in fasted and fed pigs, respectively [36,37].

Oral absorption of FB_1_ by passive non-ionic transcellular diffusion is very limited as FB_1_ is mainly negatively charged at the pH of the duodenum and jejunum in broiler chickens (pH = 6–7 [38]; pKa of the two tricarballylic acid functional groups of FB_1_: 3.49–5.83 and of the amine functional group: 9.53 [39]). Besides, also limited transcellular transporter mediated FB_1_ absorption has been suggested [34]. Also, paracellular transport of FB_1_ is unlikely as the tight junction complex only regulates transport of very small endogenous compounds, not of xenobiotics like mycotoxins and drugs. In this study, it was hypothesized that damage evoked by chronic DON exposure could lead to less complex tight junctions or a “leaky” epithelium thereby enhancing FB_1_ transport. Indeed, DON negatively affects intestinal integrity and morphology as described previously by our group [9], where the same batches of DON contaminated diets as in the present study were used. This chronic DON exposure causes shortened intestinal villi, leading to a decreased intestinal surface area and possibly leading to a reduced transport [9,10]. This reduced surface area could thus abolish the possible increased paracellular transport. However, the maximum plasma concentration (C_max_) was similar in chickens fed the control feed and the DON contaminated feed, respectively 0.033 ± 0.0213 µg/mL and 0.035 ± 0.0248 µg/mL. In accordance, Vudathala *et al.* [29] showed a C_max_ of 0.028 ± 0.103 µg/mL after oral administration of 2 mg [14C]FB_1_/kg BW to laying hens. Furthermore, feeding a DON contaminated diet had no effect on the area under the plasma concentration-time profile of FB_1_ from time 0 to 2 h (AUC_0-2 h_) when compared to the control group (Table 1), demonstrating that no effect of DON on body exposure to FB_1_ was observed. Since FB_1_ was not administered intravenously to the broiler chickens in the present study, the actual absolute oral F remains unknown. Therefore, the actual volume of distribution (Vd) and total body clearance (Cl) are computed by the modeling software as Vd/F and Cl/F, respectively. The volume of distribution of FB_1_ (Vd/F) was similar in both experimental groups, *i.e.*, 206.7 ± 92.37 and 234.3 ± 25.03 L/kg in the DON contaminated *vs.* control group, respectively (Table 1).

**Table 1:** Main toxicokinetic parameters of fumonisin B_1_ (FB_1_) administered as a single oral bolus of fumonisins to broiler chickens (2.5 mg FBs/kg BW, *n* = 6), after 3 weeks exposure to either a diet contaminated with deoxynivalenol (DON contaminated, contamination level: 3.12 mg DON/kg feed) or uncontaminated (control) feed. Values are presented as mean ± SD.

Toxicokinetic Parameter of FB_1_	DON Contaminated	Control
**C_max_ (µg/mL)**	0.035 ± 0.0248	0.033 ± 0.0213
**T_max_ (min)**	20 ± 5.0	20 ± 5.0
**AUC_0-t_ (µg/mL·min)**	83.5 ± 40.21	90.6 ± 54.07
**k_el_ (min^−1^)**	0.0075 ± 0.00155	0.0078 ± 0.00052
**T_1/2el_ (min)**	98.4 ± 22.74	106.2 ± 8.34
**MRT (min)**	150.8 ± 35.52	165.5 ± 48.81
**Vd/F (L/kg)**	206.7 ± 92.37	234.3 ± 25.03
**Cl/F (mL/min·kg)**	1544.3 ± 807.33	944.5 ± 387.33
**Rel F (%)**	92.2	100

C_max_ = maximal plasma concentration; T_max_ = time to maximal plasma concentration; AUC_0-t_ = area under the plasma concentration-time curve from time 0 to 2 h; k_el_ = elimination rate constant; T_1/2el_ = elimination half-life; MRT = mean residence time; Vd/F = volume of distribution divided by the absolute oral bioavailability; Cl/F = clearance divided by the absolute oral bioavailability; Rel F = relative oral bioavailability.

In order to compare the Vd and Cl between poultry species, the values obtained for ducks and turkeys by Tardieu *et al.* [27,28] have been divided by their reported absolute F as well. The Vd/F of broiler chickens was higher compared to ducks and turkeys, namely 74.1–85.8 and 72.3 L/kg, respectively. The clearance (Cl/F) of FB_1_ obtained after oral administration in broiler chickens was similar in both experimental groups (Table 1), and was comparable to ducks (739–835 mL/min/kg) but was higher compared to turkeys (234 mL/min/kg) [27,28]. Consequently, the elimination half-life (T_1/2el_) was twice as long in turkeys (214 min) [28] compared to broilers (106 min) and ducks (70 min) [27]. The mean residence time (MRT) was 150.8 ± 35.52 min and 165.5 ± 48.81 min in the DON contaminated group and the control group, respectively. These results are comparable with ducks (188–200 min) [27], but shorter compared to turkeys (408 min) [28]. This study also showed, in accordance to reports in other poultry species [27,28,29], low plasma levels of FB_1_ (low ng/mL range) despite the high administered dose (2.5 mg FBs/kg BW). This low oral bioavailability suggests that the systemic exposure to this mycotoxin can therefore be enhanced when the intestinal barrier and integrity is compromised [17,18].

As mentioned before, no significant differences between both groups (control or DON contaminated) could be observed for any of the toxicokinetic parameters (Table 1). Also, DON and its major metabolite, de-epoxydeoxynivalenol (DOM-1), were not detected in plasma in the present study. This is in accordance to Osselaere *et al.* (2012) [17,18] where, after three-week exposure of broiler chickens to 7.5 mg DON/kg feed, no plasma levels of DON or DOM-1 were detected above the limit of quantification (LOQ = 1 ng/mL). It has been shown that DON also selectively modulates the activities of different intestinal transporter proteins for nutrients, and negatively influences the sodium associated amino acid co-transport for serine and proline [40,41,42]. 

Although in literature it has been demonstrated that DON negatively affects the intestinal barrier function, morphology and transporter mediated nutrient transport in different animal species, chronic exposure to concentrations respecting the European maximum guidance levels in feed did not affect the oral bioavailability of FB_1_ administered as a single bolus in broiler chickens.

## 3. Experimental Section 

### 3.1. Chemicals, Products and Reagents

DON (25.9 mg DON/g culture material) and FBs (14.07 mg FB_1_/g and 4.3 mg FB_2_/g culture material) were produced *in vitro* from cultures of *F. graminearum* (DSMO 4258) and *F. verticillioides* (M-3125) [43,44], respectively, and subsequently purified and crystallized [44,45] (Romer Labs, Tulln, Austria). The standards of DON and FB_1_ for the analytical experiments were purchased from Fermentek (Jerusalem, Israel), and Sigma-Aldrich (Bornem, Belgium) for DOM-1. Internal standards (IS) for DON, ^13^C_15_-DON, and for FB_1_, ^13^C_34_-FB_1_, were purchased from Romer Labs (Tulln, Austria). The standards were stored at ≤−15 °C. Water, methanol and acetonitrile (ACN) were of LC-MS grade and were obtained from Biosolve (Valkenswaard, The Netherlands). Glacial acetic acid and formic acid were of analytical grade and obtained from VWR (Leuven, Belgium). Millex^®^-GV-PVDF filter units (0.22 µm) were obtained from Merck-Millipore (Diegem, Belgium).

### 3.2. Feed Preparation and Experimental Diets

Chickens were fed a starter diet during the first eight days of the experiment, and subsequently a grower diet until the end of the trial (day 21). These feeds are further referred to as control diets. The feed composition was described previously in detail [9,46]. Briefly, the diet was wheat and rye based, with soybean meal as main protein source during the first 16 days. From day 17 onwards, the same grower diet was fed with the exception that fishmeal replaced soybean meal as main protein source. Screening of the control feeds for contamination with mycotoxins was performed by a LC-MS/MS method, as described by Monbaliu *et al.* [47]. To produce a starter and grower diet experimentally contaminated with DON, purified crystallized DON was added to 500 g of control feed. This premix was then mixed with 5 kg of control feed to assure homogeneous distribution of the toxin. The premix was finally mixed for 20 min in the total amount of feed needed for each diet. To test the homogeneity of DON in the diets, a sample was taken at three different locations in the batch and analyzed for DON as described for the control diets.

Different tested mycotoxins, their limit of detection (LOD) and limit of quantification (LOQ) were as previously described by Antonissen *et al.* [9]. Trace amounts of FB_1_ were detected in the control feed and the contaminated feed, but the mean level of 64 µg/kg feed was below the LOQ (116 µg/kg). The levels of DON and all other tested mycotoxins in the different batches of control feed were below the LOQ. The average level of DON in the different batches of contaminated feed was 3.12 ± 0.234 mg DON/kg feed, which is below the EU maximum guidance level of 5 mg DON/kg feed for poultry [35]. The contaminated feed contained also 0.020 ± 0.007 mg 3-acetyl DON/kg feed and 0.038 ± 0.031 mg 15-acetyl DON/kg feed.

### 3.3. Animal Experiment

Twelve one-day old broiler chickens of mixed gender (Ross 308) were randomly allocated to two different groups of six birds (3♂/3♀). An 18 h/6 h light/darkness program was applied. The environmental temperature was adjusted to the changing needs of the animals according to their age. The birds of one group were fed uncontaminated feed *ad libitum* (control group) whereas the birds of the other group were fed the DON contaminated feed (DON contaminated group). Drinking water was provided *ad libitum* during the entire experiment. Feed intake was measured daily per group. The BW of all animals was measured on day 1, 8, 15 and day 21. After three weeks, chickens were fasted overnight (8 h), and subsequently all birds were administered 2.5 mg FB_1_ + FB_2_/kg BW as an intra-crop bolus. The 2.5 mg FBs/kg BW corresponded with 1.91 mg FB_1_/kg BW and 0.59 mg FB_2_/kg BW. Blood was sampled by direct venipuncture from the leg vein (*vena metatarsalis plantaris superficialis*) into heparinized tubes before (0 min) and at different time points after administration, *i.e.*, 10, 20, 30, 40, 50, 60 and 240 min. No feed was provided during the toxicokinetic experiment. Blood samples were centrifuged (2851× *g*, 10 min, 4 °C) and plasma was stored at ≤−15 °C until analysis. At the end of the experiment, all the animals were euthanized and a macroscopic post-mortem examination was carried out to reveal a possible pathology. 

The animal experiment was approved by the Ethical Committee of the Faculty of Veterinary Medicine and Bioscience Engineering of Ghent University (EC 2012/075).

### 3.4. Quantification of DON, DOM-1 and FB_1_ in Plasma

Two LC-MS/MS methods were used to quantify DON and DOM-1, and FB_1_ in the plasma samples, based on Devreese *et al.* [48]. The sample preparation procedure was the same for both methods. In brief, to 250 µL of plasma 12.5 µL of both IS and 750 µL of ACN were added, followed by a vortex mixing (15 s) and centrifugation step (8517× *g*, 10 min, 4 °C). Next, the supernatant was transferred to another tube and evaporated using a gentle nitrogen (N_2_) stream (45 ± 5 °C). The dry residue was reconstituted in 200 µL of water/methanol (85/15, *v/v*). After vortex mixing (15 s), the sample was passed through a Millex^®^ GV-PVDF filter (0.22 µm) and transferred into an autosampler vial. An aliquot (5 µL) was injected onto the LC-MS/MS instrument. The LC system consisted of a quaternary, low-pressure mixing pump with vacuum degassing, type Surveyor MSpump Plus and an autosampler with temperature controlled tray and column oven, type Autosampler Plus, from ThermoFisher Scientific (Breda, The Netherlands). Chromatographic separation was achieved on a Hypersil Gold column (50 mm × 2.1 mm i.d., dp: 1.9 µm) in combination with a guard column of the same type (10 mm × 2.1 mm i.d., dp: 3 µm), both from ThermoFisher Scientific. A gradient elution program was performed with 0.1% glacial acetic acid (DON, DOM-1) or 0.1% acetic acid (FB_1_) in water and methanol as mobile phases. The LC column effluent was interfaced to a TSQ^®^ Quantum Ultra triple quadrupole mass spectrometer, equipped with a heated electrospray ionization (h-ESI) probe operating in the negative ionization mode for DON and DOM-1, and in the positive mode for FB_1_ (all from ThermoFisher Scientific). Following selected reaction monitoring (SRM) transitions were monitored and used for quantification: for DON *m/z* 355.2 > 265.1 and 355.2 > 295.1, for DOM-1 *m/z* 339.1 > 59.1 and 339.1 > 249.0, for ^13^C_15_-DON *m/z* 370.2 > 279.1 and 370.2 > 310.1, for FB_1_ *m/z* 722.3 > 333.9 and 722.3 > 352.4 and for ^13^C_34_-FB_1_ 756.4 > 356.2 and 756.4 > 374.2. The LOQs of DON, DOM-1 and FB_1_ were 1, 2 and 1 ng/mL, respectively, whereas the LODs were 0.05, 0.04 and 0.08 ng/mL, respectively. 

### 3.5. Toxicokinetic and Statistical Analysis

Toxicokinetic analysis was performed with WinNonlin 6.3 following a non-compartmental model (Pharsight, St. Louis, MO, USA). The most important toxicokinetic parameters of FB_1_ were calculated: maximal plasma concentration (C_max_), time to maximal plasma concentration (T_max_), area under the plasma concentration-time curve from time 0 to 2 h (AUC_0-t_), elimination rate constant (k_el_), elimination half-life (T_1/2el_), mean residence time (MRT), volume of distribution divided by the absolute oral bioavailability (Vd/F), and clearance divided by the absolute oral bioavailability (Cl/F). The relative oral bioavailability (Rel F) was calculated according to the following formula:

Rel F = AUC_0-t_ DON contaminated/AUC_0-t_ control (1)

Statistical analysis was done using a Student’s *t*-test (SPSS 20.0, IBM, Chicago, IL, USA). The significance level was set at 0.05.

## 4. Conclusions

Previous literature reports have shown that DON impairs the intestinal morphology, integrity and transporter mediated nutrient transport, both *in vitro* and *in vivo*. Therefore, it was hypothesized that chronic exposure to DON could influence the oral bioavailability of FBs in broiler chickens, leading to altered exposure and toxic effects of this mycotoxin. In the present study, no significant effects on the main toxicokinetic parameters and oral bioavailability of FB_1_ after a single oral bolus administration in broiler chickens were found after chronic exposure to DON. 
